# Early agriculture and crop transitions at Kakapel Rockshelter in the Lake Victoria region of eastern Africa

**DOI:** 10.1098/rspb.2023.2747

**Published:** 2024-07-10

**Authors:** Steven T. Goldstein, Natalie G. Mueller, Anneke Janzen, Christine Ogola, Rita Dal Martello, Ricardo Fernandes, Sophia Li, Victor Iminjili, Sara Juengst, Anthony Odera Otwani, Elizabeth A. Sawchuk, Ke Wang, Emmanuel Ndiema, Nicole Boivin

**Affiliations:** ^1^ Department of Anthropology, University of Pittsburgh, WWPH 3302, S. Bouquet St, Pittsburgh, PA 15260, USA; ^2^ Department of Anthropology, Washington University in St. Louis, McMillan Hall, 1 Brookings Dr, Saint Louis, MO 63130, USA; ^3^ Department of Anthropology, University of Tennessee, Knoxville, TN, USA; ^4^ Department of Archaeology, National Museums of Kenya, Nairobi, Kenya; ^5^ Department of Asian and North African Studies, Ca’Foscari University of Venice, Venice, Italy; ^6^ Department of Archaeology, Max Planck Institute of Geoanthropology, Jena, Germany; ^7^ Faculty of Archaeology, University of Warsaw, Warsaw, Poland; ^8^ Faculty of Arts, Masaryk University, Brno, Czech Republic; ^9^ School of Archaeology, Climate Change and History Research Initiative, Princeton University, Princeton, NJ 08544, USA; ^10^ Department of Anthropology, University of North Carolina at Charlotte, Charlotte, NC, USA; ^11^ Kakapel National Monument, National Museums of Kenya, Amagoro, Kenya; ^12^ Cleveland Museum of Natural History, Cleveland, OH, USA; ^13^ Department of Anthropology, Stony Brook University, Stony Brook, NY, USA; ^14^ School of Life Sciences, Fudan University, Shanghai, People's Republic of China; ^15^ Department of Earth Sciences, National Museums of Kenya, Nairobi, Kenya; ^16^ School of Social Science, University of Queensland, Brisbane, Australia; ^17^ Griffith Sciences, Griffith University, Brisbane, Australia

**Keywords:** *Eleusine coracana*, *Vigna unguiculata*, *Sorghum bicolor*, East Africa, archaeology, agriculture

## Abstract

The histories of African crops remain poorly understood despite their contemporary importance. Integration of crops from western, eastern and northern Africa probably first occurred in the Great Lakes Region of eastern Africa; however, little is known about when and how these agricultural systems coalesced. This article presents archaeobotanical analyses from an approximately 9000-year archaeological sequence at Kakapel Rockshelter in western Kenya, comprising the largest and most extensively dated archaeobotanical record from the interior of equatorial eastern Africa. Direct radiocarbon dates on carbonized seeds document the presence of the West African crop cowpea (*Vigna unguiculata* (L.) Walp) approximately 2300 years ago, synchronic with the earliest date for domesticated cattle (*Bos taurus*). Peas (*Pisum sativum* L. or *Pisum abyssinicum* A. Braun) and sorghum (*Sorghum bicolor* (L.) Moench) from the northeast and eastern African finger millet (*Eleusine coracana* (L.) Gaertn.) are incorporated later, by at least 1000 years ago. Combined with ancient DNA evidence from Kakapel and the surrounding region, these data support a scenario in which the use of diverse domesticated species in eastern Africa changed over time rather than arriving and being maintained as a single package. Findings highlight the importance of local heterogeneity in shaping the spread of food production in sub-Saharan Africa.

## 1. Introduction

In sub-Saharan Africa, food production based on the management of domesticated cattle, sheep and goat spread before, and apparently independent from, transitions to plant agriculture [1–3]. Research on early food production across the sub-continent has focused largely on early herding, and for many regions, little is known about transmissions of domesticated crops or the origins of farming economies. Archaeological and historical data show that by 1000 years ago, agricultural systems across eastern and southern Africa [3–7] included *Sorghum bicolor* (L.) Moench (hereafter sorghum), originally domesticated in northeastern Africa, *Eleusine coracana* (L.) Gaertn (hereafter finger millet) from the eastern African highlands, and crops from West Africa including pearl millet (*Pennisetum glaucum* (L.) R. Br.) and cowpea (*Vigna unguiculata* (L.) Walp) [3–7]. More recent introductions of Asian and western hemisphere crops further diversified African farming [4,8,9]. Determining when and how plants domesticated in western, northern, and eastern Africa first spread into sub-Saharan regions, and when they were integrated with herding, requires the recovery of large assemblages of preserved ancient seeds. Outside of the Horn of Africa [10] and eastern African coast [4,11,12], recovery of crop remains of any period has been extremely limited. Currently, the earliest examples of domesticated plant foods in equatorial eastern Africa are recent (*ca* 1300 calibrated (cal.) BP for sorghum and cowpea [4]) or indirectly dated (assumed *ca* 1500 cal. BP for pearl millet, sorghum and cowpea, *ca* 1000 cal. BP for finger millet [5]). The paucity of crop recovery and direct dating impedes our understanding of when and by what routes domesticated plants spread across Africa and the timing of landrace divergences. Most importantly, data gaps limit Africa’s contributions to global debates surrounding plant domestication, the origins of agriculture and the anthropogenic impacts of early farming.

**Figure 1.**
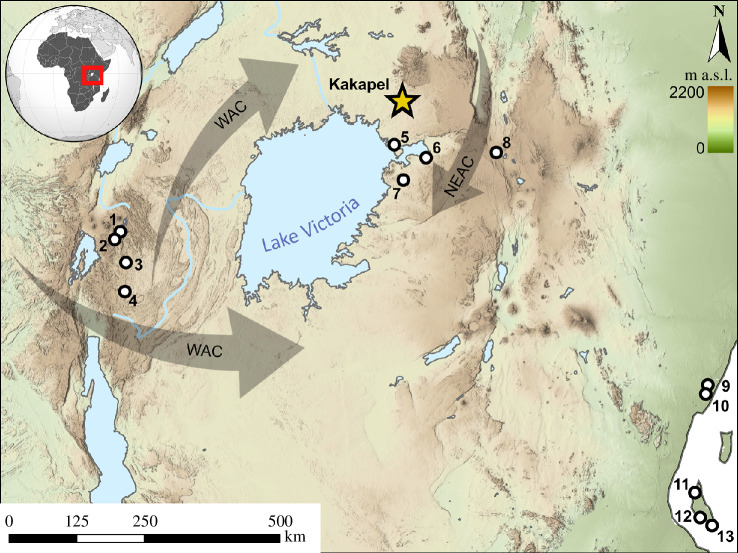
Location of Kakapel Rockshelter relative to hypothesized routes for initial spread of western African crops (WAC) and northeastern African crops (NEAC) and other sites in the region with early evidence for domesticated plants and/or referenced in text; (1) Nguri Cave; (2) Musanze; (3) Karama; (4) Kabusanze; (5) Usenge 3; (6) Wadh Lang’o; (7) Gogo Falls; (8) Deloraine Farm; (9) Panga ya Saidi; (10) Mgombani; (11) Fukuchani; (12) Unguja Ukuu and(13) Kuumbi Cave.

Several hypotheses for the spread of agriculture in eastern and southern Africa remain deeply intertwined with models for population migration. Competing models propose the introduction of farming by early herders from northeastern Africa [13,14], by Bantu-language-speaking farmers migrating from Central Africa as part of the ‘Bantu Expansion’ [7,15,16], or through interactions among economically and socially heterogeneous communities [4,17]. While the timing of crop introductions remains debated, the Lake Victoria Basin is often recognized as a key coalescence zone for diverse crops and agricultural systems that subsequently expanded southward [4,8,18–20]. Recent ancient DNA (aDNA) research also recognized this part of eastern Africa as a conduit for the major population movements possibly associated with crop transmissions [21–25]. Linking new insights on population history generated from aDNA research with high-resolution archaeobotanical records presents an opportunity to finally test longstanding hypotheses. Research at Kakapel Rockshelter, in the Chelelemuk Hills of the northeastern Lake Victoria Basin has produced a large and well-dated archaeobotanical record spanning occupations by multiple genetically distinct populations of humans (figure 1). Archaeogenetic evidence shows initial use of the site by eastern African foragers from *ca* 9000−3900 cal. BP, followed by two distinct Iron Age populations from *ca* 1000 to 300 years ago [22]. While not reflected in human aDNA at Kakapel, genetic, linguistic and archaeological evidence from the region indicate additional migrations of people with western African ancestry associated with the ‘Bantu Expansion’ through the Lake Victoria Basin around 2500 years ago [4,21,26–28].

We report here on the archaeological strata and related archaeobotanical remains from Holocene forager, early Iron Age and Late Iron Age deposits at Kakapel Rockshelter. These datasets, in concert with regional records, enable direct testing of hypothesized relationships between human and crop dispersals in the development of eastern African agricultural systems. With a total of 2320 complete seeds or fruits, of which 772 seeds are from domesticated species, the Kakapel assemblage constitutes both the largest and best-dated archaeobotanical record from the interior of equatorial eastern Africa. Importantly, this assemblage includes the earliest evidence of domesticated crops in this region in the form of the legume crop cowpea, which was probably domesticated in western Africa, where the earliest archaeological evidence has been found [29,30]. Supplemented by archaeological and zooarchaeological analyses, the unique combination of multi-phase archaeobotanical and aDNA datasets provides critical new insight into the timing and mechanisms for the arrival of other plant and animal domesticates from across the African continent. These findings highlight the importance of the Lake Victoria Basin as a coalescence zone for diverse populations with novel food-producing strategies, contributing to a new understanding of the emergence of farming economies that would come to characterize much of sub-Saharan Africa into the present day [4,13,31,32].

## 2. Material and methods

### (a) Kakapel Rockshelter

Kakapel is a granitic inselberg within the Chelelemuk Hills in North Teso, Busia County, in western Kenya. The pluton is located at around 1420 m a.s.l. at the base of Mt. Elgon, just outside the margins of the Lake Victoria Basin drainage. This is a region with one of the highest rainfalls of Kenya, receiving an average of 1250−2000 mm of precipitation distributed across two rainy seasons per year. Several Holocene sites around the Lake Victoria Basin demonstrate transitions from the Later Stone Age fisher-forager communities, who produced distinctive ‘Kansyore’ ceramics, to the Early Iron Age ‘Urewe’ groups associated with Bantu-speaking migrants [20]. Gogo Falls and Wadh Lang’o have intermediate herder occupations *ca* 2000−1600 cal. BP southeast of Kakapel [20,33,34], and early mobile herders are now documented in the highlands 140 km to the east [35]. Kakapel is therefore situated in an intermediary zone where farming, herding and foraging lifeways intersected at the start of the Iron Age.

Kakapel Rockshelter was first described by Osaga Odak in 1977 as a rock-art locality associated with Late Iron Age potsherds within a shallow shelter formed by erosion of the granitic pluton [36,37]. Owing to its well-preserved rock art, Kakapel was accessioned as a national monument. The National Museums of Kenya began test excavations in 2012, revealing earlier deposits and human burials. Subsequent research from 2018 to 2020 by this team focused on archaeobotanical recovery and documentation of the stratified archaeological sequence. Excavations were concentrated in four trenches (24 m^2^ total) with most botanical and faunal material recovered from trenches II and III, within the protected area of the shelter (electronic supplementary material, figures S1–S3). Owing to the high density of archaeological features including hearths, pits and refuse deposits (electronic supplementary material, figures S4 and S5), excavations followed a single-context method following natural sediment changes when detected, with 5 cm arbitrary subdivisions within undifferentiated strata. All artefact finds over 2 cm, context boundaries and elevations, and both top and bottom points for flotation samples for the main occupational horizons were digitally mapped and recorded using a Leica total station.

### (b) Archaeobotanical recovery, analysis and dating

Over the course of two field seasons, 135 flotation samples were taken at Kakapel, giving a total soil volume of 1430 l. Of these, 113 samples (1156 l of sediment) have been analysed (table 1). We took samples from contexts with visible charcoal inclusions, or that were of particular interest (e.g. burials and pit features), as well as arbitrary samples for each context. We collected samples by ‘chunking out’ (not trowelling, as this would damage fragile carbonized plant materials) 10 l of sediment from contexts of interest, or the entire context if < 10 l remained. These soil samples were processed by bucket flotation. The light fraction was collected in 355 μm mesh-lined fabric bags and line dried, then stored in hard containers for transport/export. The heavy fraction was sieved through 2 mm geological sieves and sorted in the field. Charcoal in the heavy fraction was combined with the > 2 mm light fraction. Other artefacts in the heavy fraction were weighed and counted and are curated at the National Museums of Kenya in Nairobi.

**Table 1.** Summary of archaeobotanical sampling at Kakapel Rockshelter.

phase	samples	litres of sediment	seeds	seeds l^−1^	crop seeds l^−1^	non-crop seeds l^−1^	nutshell/wood (g)
phase I	40	412	310	0.75	0.43	0.32	0.035
phase II	18	171.5	488	2.72	2.04	0.68	0.034
phase III	37	383.5	1171	2.95	2.10	0.85	0.020
phase IV	11	109	209	1.84	0.21	1.63	0.002
unknown or mixed	8	80	142	1.76	0.17	1.59	0.101
total	113	1156	2320				

Archaeobotanical samples were analysed at Washington University in St Louis, USA, and at the Max Planck Institute for the Science of Human History in Jena, Germany. All material was inspected under a dissecting microscope at 10–40 × magnification. Material greater than 2 mm was sorted completely into categories: wood charcoal, nutshell, seeds, unidentifiable carbonized material and uncarbonized material. Material > 1 mm to > 0.425 mm was scanned for seeds and seed fragments. All complete seeds were identified to family or described and given a numbered type (comprehensive identifications of wild taxa are forthcoming). Seed identifications were made with reference to standard seed manuals [38] and comparative materials housed in the East Africa Herbarium, Missouri Botanical Garden Herbarium, Cornell L. H. Bailey Hortorium, or obtained from the United States Germplasm Research Information System. Morphometric data for finger millet and cowpea specimens were collected using digital microphotographs of the ventral surface of seeds and analysed in ImageJ software [39].

Larger nutshell and seed samples were dated using standard radiocarbon pre-treatment protocols and procedures; however, attempts to date single grains of small-seeded finger millet failed using standard approaches, as not enough carbon remained following full acid-base-acid (ABA) pre-treatment. Successful direct dates on individual finger millet grains weighing 0.7–1.0 mg each were obtained by the National Ocean Sciences Accelerator Mass Spectrometry Facility (NOS-AMS), Woods Hole, USA, using an acid-only pretreatment [40,41]. A pooled sample of several finger millet grains was dated using the complete ABA pretreatment and provided radiocarbon measurements with distributions overlapping with a single grain from the same feature dated using acid-only pretreatment. Given the relatively young age of the samples, this demonstrates minimal contamination and demonstrates the reliability of direct small-sample finger millet dates from Kakapel deposits. Radiocarbon measurements were calibrated using the software OxCal v. 4.4 and SHCal20, the radiocarbon calibration curve for the southern hemisphere [42,43].

### (c) Zooarchaeology and zooarchaeology-by-mass-spectrometry analyses

A total of 6902 g of faunal remains were recovered. All identifiable specimens, as well as non-identifiable specimens over 20 mm in maximum dimension, were analysed. Each specimen was identified to the narrowest taxonomic category, using the comparative faunal collections at the National Museums of Kenya. Description of the assemblage is ongoing; however, the initial analysis was focused on identification of the domesticated species (i.e. cattle, goat and sheep) that were introduced by pastoralist migrations from the northeast [1,21] and so are relevant to reconstructing broader agricultural systems. Given the highly fragmentary nature of the faunal assemblage, no bones were complete enough to allow definitive identification to a domesticated species based on morphology, so we used zooarchaeology and zooarchaeology-by-mass-spectrometry (ZooMS), which uses characteristic peptide sequences of bone collagen to identify animal taxa from bone fragments.

Faunal bone fragments tentatively identified as *Bos* (cattle) and *Capra* (goat genus) were selected for ZooMS analysis along with a diverse sample of non-identifiable fragments with good cortical bone preservation, which have a higher likelihood of protein recovery. In total, 52 bone fragments were sampled for ZooMS. An approximately 20 mg bone chip from each sampled specimen was analysed using established protocols [44]. Samples were first demineralized in 500 µl of 0.5 M hydrochloric acid at 4°C for 2 days. The supernatant was removed and the samples were rinsed twice with 50 mM ammonium bicarbonate before incubation at 65°C for 1 h. Then, 50 µl of the resulting supernatant was treated with 0.2 µg trypsin (ThermoFisher Pierce Trypsin Protease) and incubated at 37°C for 18 h. The resulting solution was subjected to a C18 cleanup (ThermoFisher Pierce C18 Tips) and was mixed with a matrix solution of α-cyano-4-hydroxycinnamic acid (10 mg ml^−1^ in 50% acetonitrile (ACN)/0.1% trifluoroacetic acid (TFA). The samples were spotted onto a target plate and allowed to co-crystalize. The analysis was carried out using a Bruker Autoflex Speed LRF (Bruker Daltonics) and the resulting spectra were compared with reference libraries [44–48] using FlexAnalysis software. Taxa discussed for each phase below represent the results of both morphological and ZooMS-assisted identifications (electronic supplementary material, tables S3 and S4).

## 3. Results

Extensive flotation efforts produced an exceptionally large assemblage of archaeobotanical remains from multiple archaeological horizons, and direct dating of identified plant and animal remains has produced a high-resolution radiocarbon chronology for site occupation and introduction of various domesticated species (table 2). Integrating stratigraphic horizons, three-dimensional point data, material culture patterns, aDNA findings [22] and direct radiocarbon dates on human, animal and plant remains allowed us to identify four distinct occupational phases. The four temporal phases reflect occupation by: (I) ceramic-producing fisher-foragers of the Kansyore tradition from *ca* 9000 to 3900 cal. BP; (II) Early Iron Age farmers with crops from western Africa and Urewe-style ceramics, from *ca* 2300 to 1800 cal. BP; (III) Later Iron Age agropastoralists associated with Nilotic-speaking populations *ca* 1200−300 cal. BP and; (IV) a second expansion of Nilotic peoples associated with historic migration of Teso-speakers. Archaeobotanical material from contexts where either a definitive phase could not be attributed, or where there was suspected disturbance/mixing between phases, were not considered in the reconstructions of agricultural systems below. These included eight out of the 113 samples analysed, or 80 of 1156 l of analysed sediment, from which 142 seeds were recovered (table 1).

**Table 2.** Radiocarbon dates from Kakapel Rockshelter calibrated with SHCal20 [43,49,50].

					calibrated date BP	
	archaeological period	14C laboratory code	material/species	14**C years bp**	68.2%	2 sigma
aDNA samples	phase IV	SUERC-86058^b^	*H. sapiens*	222 ± 29	305–151	311–100
	phase III	SUERC-86059^b^	*H. sapiens*	895 ± 28	898–738	906–731
	phase I	SUERC-86057^b^	*H. sapiens*	3584 ± 28	3957–3840	3976–3777
plant and animal samples	phase IV	OS-167169	*Pisum*	210 ± 15	293–152	300–101
	phase III	WK-47412	*S. bicolor*	848 ± 16	772–727	785–695
	phase III	OxA-38796	*S. bicolor*	864 ± 21	775–733	897–723
	phase III	OxA−40174	*B. taurus*	901 ± 18	899–747	904–734
	phase III	OS-167170	*Pisum*	920 ± 20	904–786	911–752
	phase III	Wk-48697^a^	*S. bicolor*	940 ± 18	908–794	913–792
	phase III	Wk-48699	*Fabaceae*	962 ± 18	917–802	923–794
	phase III	OS-162692	*E. coracana*	965 ± 25	919–800	925–793
	phase III	OS-174285^c^	*E. coracana*	975 ± 15	923–829	925–798
	phase III	OS-174281	*E. coracana*	985 ± 20	928–829	953–797
	phase III	OS-174283^c^	*E. coracana*	985 ± 15	928–831	930–799
	phase III	OS-174284^c^ ^d^	*E. coracana*	995 ± 20	954–831	957–800
	phase III	OS-166571	*E. coracana*	1020 ± 20	955–921	959–914
	phase III	OS-174282^c^	*E. coracana*	1020 ± 20	955–921	959–914
	phase III	OS-162716^a^	*E. coracana*	1060 ± 35	1049–925	1058–918
	phase II	WK-47413	indeterminate nutshell	1713 ± 15	1688–1568	1693–1545
	phase II	OxA-40173	*B. taurus*	2235 ± 20*	2320–2159	2331–2155
	phase II	Wk-48695	indeterminate nutshell	2236 ± 18	2320–2160	2331–2155
	phase II	SUERC-98421	indeterminate nutshell	2266 ± 29	2340–2182	2345–2156
	phase II	OS-168214^a^	*V. unguiculata*	2280 ± 20	2344–2215	2348–2180
	phase I	OxA-38811	indeterminate nutshell	6453 ± 26	7422–7331	7425–7320
	phase I	SUERC-90741	*C. schweinfurthii*	6575 ± 25	7486–7429	7560–7427
	phase I	OxA-38809	indeterminate nutshell	8197 ± 28	9259–9030	9274–9025

^a^ Earliest occurrence of the domesticated species at Kakapel.

^b^ Date reported in [22].

^c^ Recovered from strata associated with phase I.

^d^ Combined sample of 6 finger millet grains with ABA pretreatment.

### (a) Phase I

Human presence at Kakapel begins with the appearance of diagnostic Kansyore forager pottery (electronic supplementary material, figure S6) in contexts dated to *ca* 9000 cal. BP; one of the earliest dates for Kansyore material in the Lake Victoria Basin [51–53]. Kansyore presence terminates *ca* 3900 cal. BP based on the direct dates from a young adult male buried in a pit feature [22]. Phase I levels contained numerous charcoal-rich features from informal burning events and hearths which yielded a total of 301 seeds, 264 g of wood charcoal and 9 g of nutshell. The nutshell-to-wood charcoal ratio (0.035) was slightly higher for samples from phase I than for those from later phases, perhaps suggesting foragers at Kakapel relied more on forest resources. In this metric, charcoal is used as a norming value that represents the likelihood of preservation through carbonization at the site; an increase in nutshell relative to charcoal thus represents more intensive use of foods foraged from trees and shrubs [54]. Seeds, presumably wild, from the following families were recovered: Amaranthaceae, Asteraceae, Caryophyllaceae, Convolvulaceae, Fabaceae, Oxalidaceae, Portulacaceae, Rosaceae and Solanaceae, along with numerous unidentified types, which are still being analysed.

Some of these families include both woody and herbaceous species in Kenya (see electronic supplementary material, table S2) so their interpretative value is limited until more specific identifications are made. Other families are dominated by herbaceous plants globally, with no woody taxa or lianas currently occurring in Kenya: for phase I, these are Asteraceae, Caryophyllaceae and Oxalidaceae [55]; (electronic supplementary material, table S2). Also, although one woody taxa of Amaranthaceae does occur locally [55], all Amaranthaceae seeds from Kakapel belong to *Amaranthus* L. sp., a genus of annual or short-lived perennial herbs. There are several species of *Amaranthus* sp. in Kenya, and they are common field weeds and/or encouraged as leafy greens at present in the region [56]. The presence of these taxa indicates at least some ancient forest disturbance around the site.

Domesticated finger millet grains were present in 25 of 40 phase 1 flotation samples accounting for 42% of the seeds recovered. The seeds of the wild progenitor of finger millet (*E. coracana* ssp. *africana*) were also recovered from 6 of the 40 phase 1 samples. Finger millet seeds are so small that individual grains are difficult to date, yet we were able to obtain direct radiocarbon dates on several grains recovered from phase I layers. These all dated to between 1200 and 900 years ago, roughly 5000 years younger than nutshell and charcoal fragments in the same contexts (see table 2). Because there is no evidence for transposition or depositional mixing in the material culture or the radiocarbon dates for larger seeds or plant macro-remains we suspect that this reflects the high potential for vertical mobility of small seeds. Unfortunately, this means that macrobotanical remains from phase I contexts must be treated with caution until ages can be confirmed by direct radiocarbon dating.

Fauna from this phase (number of identifiable species (NISP) = 440; see electronic supplementary material, table S3) is dominated by reduncines including reedbuck (*Redunca* sp.) and bushbuck (*Tragelaphus scriptus*) and the alcelophines, hartebeest (*Alcelaphsus buselaphus*) and topi (*Damaliscus lunatus*). Wild suids, rodents and other smaller mammals were also present in low frequency. No domesticates or likely domesticates were identified in any phase I sampling. Overall, the phase I assemblage indicates a diversified foraging strategy that included resources from both forest and open habitats.

### (b) Phase II

**Figure 2.**
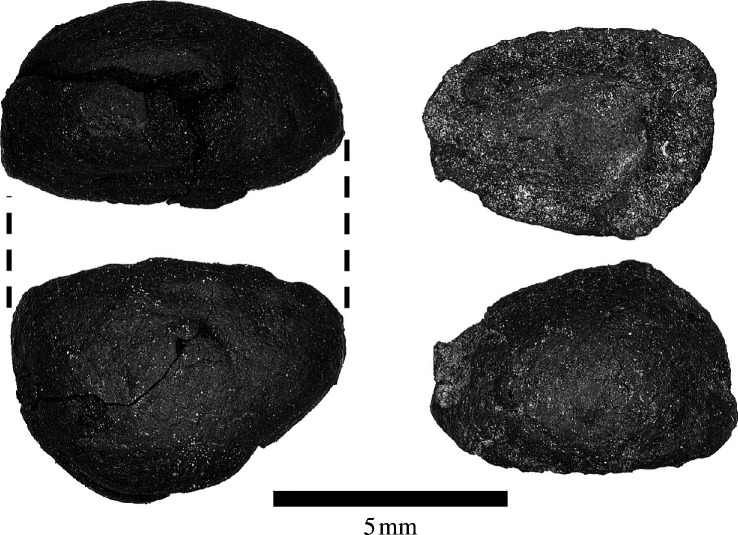
(*V. unguiculata* (L.) Walp.) specimens recovered from an Urewe-period hearth dated to phase II. Left: ventral (top) and lateral (bottom) views of a complete specimen; right: interior (top) and exterior (bottom) views of a single cotyledon.

Phase II contexts are the only deposits at Kakapel yielding diagnostic Urewe incised and dimple-based ceramics, which archaeologists often associate with arrivals of Bantu-speaking populations from the west *ca* 2500 years ago [4,57,58] (electronic supplementary material, figure S7). Phase II at Kakapel contains the earliest directly dated domesticated crop from the site: the legume cowpea (figure 2). The earliest evidence for domesticated cowpea dates to 3700 cal. BP in Ghana [29,30], indicating likely domestication in western Africa. However, its wild progenitor grows throughout sub-Saharan Africa, and modern genetics suggests the possibility of a second domestication in eastern Africa [30,59]. Cowpeas (one complete seed and three cotyledons) were recovered at Kakapel from a distinct clay-lined burning feature, interpreted as a hearth, in phase II horizons that yielded Urewe potsherds (electronic supplementary material, figure S5). One of these specimens was directly dated to 2215−2348 cal. BP (OS-168214, 2280 ± 20 radiocarbon years before present (rcybp)), consistent with two dates on nutshell fragments from the same feature (see table 2). The co-occurrence of new material culture (Urewe pottery) with a probable western African crop (cowpea) is consistent with the ‘Bantu Expansion’ hypothesis for early agriculture in this region [4,28,58,60].

**Figure 3.**
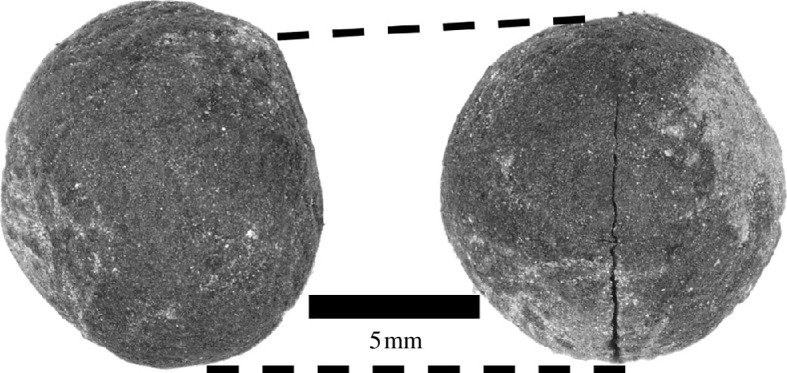
*P. sativum* L. or *P. abyssinicum* A. Braun specimen recovered from phase II hearth. Left: lateral view and right: ventral view.

However, the same hearth also yielded a single pea (*Pisum* sp.), a crop with its origins to the north (figure 3). To our knowledge, this is the only evidence of peas in Iron Age eastern Africa. The specimen lacks a seed coat and is morphologically ambiguous between Mediterranean field pea (*Pisum sativum* L.) and the closely related crop *dekoko*, or Abyssinian pea (*Pisum abyssinicum* A. Braun)*,* an endemic Ethiopian crop with a complicated evolutionary history [61,62]. Hereafter, we will refer to *Pisum* sp. specimens by the common name 'pea'. Peas may have dispersed through the Nile in the early Holocene, and the species is documented both in Ethiopia and southern Nubia in the third millennium BP [10,63]. Because it is the only pea from phase II contexts, this specimen has not been directly radiocarbon dated (other pea samples from phase III have been dated; see table 2 and electronic supplementary material, table S2). While we recognize that this is not ideal given the potential for bioturbation, the pea was recovered from a particularly well-defined hearth feature (electronic supplementary material, figure S5) that yielded three consistent early Iron Age dates (table 2). An early Iron Age introduction of the crop, therefore, remains possible and requires further investigation.

In addition to the domesticated legumes, 125 finger millet grains were recovered from phase II contexts. Four came from the hearth feature where the cowpeas and peas were recovered, but even as a pooled sample, these were not heavy enough to directly date. A greater concentration of finger millet seeds from a phase II context came from an ashy area just adjacent to this feature (*n* = 46, plus 85 probable finger millet grains). Unfortunately, our attempts to directly date these specimens were not successful, despite a date on nutshell from the same feature of 2320‒2160 cal. BP (2236 ± 18 rcybp, Wk-48695). Considering the late dates from finger millet recovered from phase I (table 2), we are not able to confidently attribute the grains from these features to phase II. To summarize, both finger millet and a pea were recovered from a secure hearth feature (electronic supplementary material, figure S4) yielding several consistent Early Iron Age dates; however, the earliest successful direct dates for both crops are later (see table 2).

Phase II samples were also rich in the seeds of presumably wild plants, including those from the families Amaranthaceae, Asteraceae, Convolvulaceae, Fabaceae, Malvaceae, Poaceae, Portulaceae, Polygonaceae and Rosaceae, in addition to numerous unidentified types that are still being analysed. Again, not all family-level identifications are useful for reconstructing the diet and environment, due to the diversity of taxa they encompass. For phase II, recovered seeds from families that do not include any woody taxa or lianas in Kenya include Amaranthaceae, Asteraceae and Poaceae (electronic supplementary material, table S2). In addition to the unidentified wild grass (Poaceae) seeds from phase II, 97 wild finger millet (*E. coracana* ssp. *africana* [Kenn.-O'Byrne] [64]) seeds were also recovered from these contexts. This weedy taxa indicates the presence of disturbance and open habitats near the site. However, the nutshell/wood ratio was almost identical between phases I and II (phase I = 0.035, phase II = 0.034), which also suggests the continuing importance of forest resources and/or agroforestry.

While faunal remains in phase II continue to be primarily local wild species (NISP = 134; see electronic supplementary material, tables S3 and S4), we also recovered a bone fragment of domesticated cattle (*Bos taurus*) less than 1 m away from the hearth feature that yielded cowpea, pea and finger millet specimens. ZooMS analysis of the specimen confirmed collagen protein signatures that differentiate domesticated *Bos taurus* from *Syncerus cafer* and other wild species of a similar size class [47]. Direct radiocarbon dating of the bone indicates people had access to domesticated animals by 2331‒2155 cal. BP (OxA-40173, 2235 ± 20 rcybp), a few centuries before cattle and other livestock are detected at early pastoralist sites on the southwestern Lake Victoria Basin [20,33]. At present, this constitutes the earliest direct date for cattle in this region and provides the first evidence that early farmers also had access to cattle, possibly through exchange with herders in the highlands east of the lake [35,65–67].

### (c) Phase III

**Figure 4.**
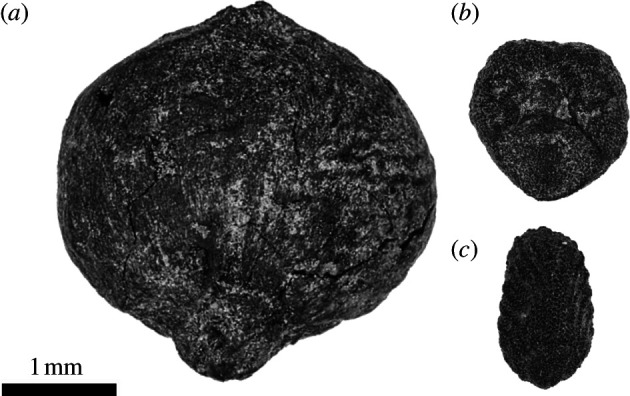
Examples of (*a*) sorghum (*S. bicolor* (L.) Moench), (*b*) domesticated finger millet (*E. coracana* (L.) Gaertn.) and (*c*) wild finger millet (*E. coracana* ssp. *africana* [Kenn.-O'Byrne] Hilu & de Wet) from phase III samples at Kakapel Rockshelter.

Phase III at Kakapel consists of dense archaeological horizons with multiple burning and midden features, pits, sealed hearths and ash dumps. These features yielded a large archaeobotanical assemblage containing three eastern and northern African crops (finger millet, sorghum (see figure 4) and peas), but from which western African grains and legumes were absent. Based on directly dated specimens, phase III inhabitants at Kakapel were cultivating finger millet, a native east African domesticate, by 1050 cal. BP (OS-166571, 1020 ± 20 rcybp). Sorghum, domesticated in northeastern Africa [68,69], first appears at Kakapel over a century later, between 908 and 794 cal. BP (Wk-48697, 940 ± 18 rcybp; OxA-47412, 848 ± 17 rcybp), and is never nearly as abundant or ubiquitous as finger millet. In total, 476 carbonized finger millet grains and 9 sorghum grains were identified from features and arbitrary sediment flots from phase III contexts. Two peas were recovered from phase III, one of which was directly dated to 911−752 cal. BP (OS-167170, 920 ± 20 rcybp).

The botanical assemblage from phase III appears to show a slight reduction in diversity compared to earlier phases. Other than crops, seeds from Amaranthaceae, Fabaceae, Poaceae, Portulaceae and Polygonaceae, as well as unidentified types, were present. Non-crop seeds were approximately as abundant as in phase II (table 1), but there were fewer types. The only taxa identified in phase III that do not include any woody taxa or lianas are Poaceae and *Amaranthus* L. sp., which are numerous (*n* = 30) (electronic supplementary material, table S2). There was a drop in the nutshell-to-wood ratio ( 0.020) in this phase, which suggests less use of forest resources and/or a switch from agroforestry to open-field agriculture. A more detailed analysis of changes in ecology and subsistence between phases must await the identification of the numerous non-crop seeds present in the assemblage.

The dominance of eastern and northern African crops in phase III may be related to a second demographic shift in the region. aDNA recovered from an isolated human tooth reflects the presence of a new population by 950 cal. BP [22]. The phase III individual at Kakapel and a roughly contemporaneous Iron Age individual sampled from the site of Deloraine in central Kenya (1170‒790 cal. BP) reflect likely admixture between new migrants with northeastern African ancestry and earlier Pastoral Neolithic herders [21,22]. The nearly simultaneous appearance of eastern and northern African crops at Kakapel in association with this new ancestry signature may suggest complex dispersals of mixed economy pastoralists into the Great Lakes Region from the east, as sorghum was already under cultivation along the Swahili Coast by 1300 cal. BP [4]. While pastoralism was widespread in eastern Africa by this time, only one domesticated cattle bone and one caprine were recovered from phase III contexts. Preliminary morphological analysis of the remaining assemblage continues to reflect the hunting of wild savanna species (NISP = 99; electronic supplementary material, table S3).

### (d) Phase IV

Phase IV horizons are thinner, near-surface, levels dominated by a mix of Later Iron Age, historical and recent pottery. Archaeological and botanical assemblages suggest a change in site use over recent centuries. Only six finger millet grains and one sorghum grain were recovered from these strata. The lack of finger millet in recent strata is somewhat surprising, given its abundance and ubiquity in earlier layers. Peas, on the other hand, are well represented in phase IV, with 15 specimens, one of which was directly dated to 300‒101 cal. BP (OS-167169, 210 ± 15 rcybp). This contrast further highlights the importance of finger millet to the livelihoods of the Iron Age people at Kakapel, especially when we consider that this species is not likely to be preserved through carbonization [70]. Seeds of Amaranthaceae, Asteraceae, Cannabaceae, Caryophyllaceae, Convolvulaceae, Cyperaceae, Fabaceae, Malvaceae, Poaceae, Portulaceae, Solanaceae and Rosaceae were also recovered from phase IV contexts, making it the most taxonomically diverse phase (electronic supplementary material, table S2). Of these, several families are indicative of open habitats, and the appearance of sedges (Cyperaceae) is notable given that they indicate wet or well-watered environments.

Phase IV also contained the burial of a well-preserved adult woman dated to 300 cal. BP, whose genetic ancestry reflects a fourth population at Kakapel, which was more closely related to Nilotic-speaking peoples from northeastern Africa [22]. This is likely to be related to recent migrations of Teso-speaking communities who currently live in the Kakapel area. Additional subsistence changes have come from the introduction and proliferation of western hemisphere and Asian crops, especially maize, which appears in microbotanical [71,72] and historical [73] records from Kenya and Tanzania by *ca* 180 years ago. We did not recover any western hemisphere crops at Kakapel, though they constitute most crops grown there today.

## 4. Discussion

Remains of cowpea at Kakapel Rockshelter directly dated to *ca* 2300 cal. BP constitute the earliest documented arrival of a domesticated crop—and presumably of farming lifeways—to equatorial eastern Africa. The assumed western African origin of cowpea [29,59] directly associated with Urewe pottery supports hypotheses for transmission of farming into the Lake Victoria Basin concurrent with the spread of Bantu-speaking peoples migrating from Central Africa as part of the ‘Bantu Expansion’ [28]. Evidence from Kakapel also provides the first hints at the kind of wild plant food economies that preceded and continued alongside the introduction of domesticates. Recovery of *Amaranthus* is particularly important in this regard. Ethnographic studies identified *Amaranthus graecizens* L. and *Solanum nigrum* L. as the most important fresh plant foods for Okiek foragers in a similar high-altitude forest environment in southern Kenya [56]. Though more precise identifications are needed, it is intriguing to consider that leafy greens, with their need for open and frequently disturbed habits, may have been an important part of forager diets prior to the spread of farming.

Phase II at Kakapel is represented only by a cluster of features concentrated in Trench 3 (electronic supplementary material, figure S1) that are probably indicative of a few short-term occupations. It remains entirely possible that peas and cowpeas were transported to Kakapel from elsewhere and attempts to grow these crops locally did not result in an immediate or lasting transition to widespread agriculture. Despite flotation efforts at other Urewe-bearing sites, no other evidence of western African crops has been reported before *ca* 1600 cal. BP (based on associated charcoal in Rwanda) [5]. Growing western African lowland-adapted crops in the eastern African highlands may have proven initially difficult as soil conditions, seasonal rainfall patterns, elevation and temperature differ markedly between west/central and eastern Africa. It is also clear from faunal records at Kakapel and elsewhere that hunting and foraging wild resources were more important for early Bantu subsistence than these imported crops [4,47,53,74,75]. In the long-term, local ecological knowledge was critical in structuring community-scale choices about which crops to adopt and how to best integrate them into existing food systems.

Recovery of two northeastern African crops (peas and finger millet) from phase II features at Kakapel prevents ruling out the possibility that multiple simultaneous crop introductions occurred through different mechanisms at the beginning of the Iron Age. Given the identification of early peas in northeastern Africa [10,63], it is possible this and other crops spread along the Nile River into the Lake Victoria region before, or simultaneously with, arrivals of west African crops. More research is needed to determine the timing of pea spread, and which groups are responsible for its transmission into eastern Africa. Until these issues can be resolved, western African crops appear to be the earliest to arrive in the Great Lakes region of eastern Africa.

Early Bantu forager-farmers may also have benefited from the incorporation of domesticated livestock, likely through exchange with mobile herding communities living in the southern Lake Victoria Basin and Uasin Gishu Plateau to the east of Kakapel [35,76]. Detection and proteomic confirmation of a cow bone from phase II (*ca* 2300 cal. BP) provides the earliest direct date for domesticated livestock in the region and indicates that Urewe-producing groups had access to cattle, possibly through exchange with nearby herders. If herders gained access to domesticated crops in return, it could have proven transformative for pastoralist strategies in eastern African grasslands. Certainly, the transmission of cattle to Bantu-speakers was critical in shaping the diversified agropastoral economies that were transmitted from the Great Lakes through later phases of the Bantu Expansion across southern Africa [6,8].

Unambiguous evidence of eastern or northern African crops—including directly dated sorghum, finger millet and pea—is present during the Late Iron Age phase III at Kakapel and may be associated with distinct migrations of Nilotic-descendent agro-pastoralists that is detected in aDNA from Kalapel *ca* 950 cal. BP and from Deloraine Farm in Central Kenya *ca* 1100 cal. BP [21,22]. Sorghum was widely cultivated along the Swahili coast centuries earlier and is assumed to have been widely grown elsewhere by this time [4], so its adoption at Kakapel may reflect local introductions by these newer communities as they shifted toward Lake Victoria from the east.

Previous reports have placed other African crops across eastern and southern Africa as early as 1700‒1400 cal. BP, but except for high-resolution research confirming pearl millet, sorghum and Asian crops at sites along the Indian Ocean coast [4] (figure 1), claims rely on indirect dates and/or problematic contexts. Sorghum, pearl millet and cowpea have been reported from a cluster of Urewe sites in Rwanda (figure 1) around 1600 cal. BP with finger millet appearing only in Late Iron Age contexts *ca* 1000 cal. BP (similar to Kakapel phase III) [5]. These are all indirect dates and so should be taken with caution, though they align with the pattern at Kakapel in that they show an earlier presence of western African crops and apparent later integration of finger millet.

Further south, sorghum and cowpea were reported from the sites of Muteteshi and Mondake near the Mulungushi River in Central Zambia, but these remains were never photographed, dated only by association and were from very shallow deposits in areas under active cultivation [77,78]. Sorghum and a single pearl millet grain have been reported from strata dated to 1500‒1200 BP from Zimbabwe and southern Africa, but these again are limited assemblages without direct dates [79]. While recent morphometric work on finger millet allows some preliminary observations of morphological change over time and diversity across space [70], more and larger assemblages of other crops are needed to assess patterns of evolutionary change as they were transmitted across the highly diverse microclimate zones of eastern Africa. Such data would contribute to understanding how and why different combinations of domesticated plants and animals spread at different times to different regions.

Crowther *et al*. [4] draw on the concept of ‘mosaics’ to explain this apparent temporal variability in the transition to food production across eastern Africa and the regional heterogeneity in the combinations of farming, herding and foraging that appear in the archaeological record. In this framework, communities developed localized food-acquisition strategies that were suited to their specific ecological conditions and cultural preferences with diverse patterns of interaction influencing shifts in these lifeways through time and across space [17,80]. Evidence from Kakapel Rockshelter builds on this model, highlighting that population migration was a critical—though not exclusive—mechanism for the translocation of African crops with disparate geographic origins. The frequent migrations documented in eastern African genetic and ethnohistoric records did not overwrite preexisting subsistence economies, but they did significantly widen the options available to communities by introducing new crops.

Continued reliance on hunting and gathering at Kakapel from the early Holocene to the historic period, despite access to domesticated plants and animals, is one example of how new and pre-existing foodways were selectively merged over time. Despite their early arrival at Kakapel, western African crops did not take hold there. Likewise, even though we recovered hundreds of seeds from Kakapel, the important west African cereal crop pearl millet is entirely absent, providing a contrasting example to the eastern African coast, where western African crops became important [4]. A millennium later, people presumably from the northeast seem to have brought a suite of crops that became staples around the Lake Victoria Basin. This new set of crops appears to correlate with more intensive occupation and agricultural activity in the area over the past 1000 years, which has further implications for understanding the pace of local anthropogenic impacts and landscape change. Migrations also catalysed new exchange relationships, stimulating the reconfiguration of subsistence economies as evidenced by the revolutionary role cattle had once adopted by Bantu-speaking communities [8,81].

Crop diversification provides communities with greater resilience to climatic uncertainty, insect predation and other risks to farming lifeways in eastern Africa [82]. Migration and expanding interaction networks were therefore critical in building and reinforcing the agricultural systems that supported large populations and state formation around the African Great Lakes prior to European colonialism. Combining archaeological, archaeobotanical, proteomic and archaeogenetic approaches demonstrates the central role of complex, overlapping population movements and migrations in the introduction and diversification of agricultural repertoires in eastern Africa.

## 5. Conclusion

Recovery of a large archaeobotanical collection from Kakapel Rockshelter in western Kenya contributes to emerging models for the spread of early agriculture in eastern Africa. The presence of domesticated cowpea *ca* 2300 years ago provides the first evidence for the arrival of farming communities to the Lake Victoria Basin and contemporaneous evidence of cattle demonstrates rapid access to livestock, possibly through exchange relationships with herders in adjacent regions. Whether or not northeastern African crops were present earlier, their abundance at Kakapel after 1000 cal. BP indicates the ongoing transformation of local agricultural systems. As with the transmission of western African crops, population migrations evident in the aDNA of people buried at Kakapel appear to have played a critical role in expanding access to, and shifting focus among, diverse domesticated resources across the eastern African landscape. Demographic changes and the resulting reformulation of interaction networks helped shape subsistence mosaics, with diverse communities selecting options for what to grow and what to acquire through trade based on local preferences and environmental circumstances. While Kakapel is only a single datapoint, it is particularly important in centring the Lake Victoria Basin as a crossroads where western African crops, northeastern African crops and livestock were first exchanged and integrated before spreading further to southern Africa. Overall, the flora and fauna recovered from Kakapel present a picture of a diverse and flexible subsistence system in which hunting and foraging were complemented, not replaced, as agricultural systems shifted over the course of multiple migrations through the region. These findings provide new foundations for understanding how and when diverse domesticated plant foods were incorporated into eastern African lifeways and demand increased attention to the study of early crop transmissions across the continent.

## Data Availability

All data reported here are available in the article main text and supplementary files [83].
